# Timing Matters: Viticultural Land Use Determines Responses in Structure and Function of Fungal Stream Communities Across One Growing Season

**DOI:** 10.1111/gcb.70085

**Published:** 2025-02-13

**Authors:** Verena C. Schreiner, Moritz Link, Gesa Amelung, Katharina Ohler, Romana Salis, Florian Leese, Ralf B. Schäfer

**Affiliations:** ^1^ Faculty of Biology University of Duisburg‐Essen Essen Germany; ^2^ Research Center One Health Ruhr University Alliance Ruhr Essen Germany; ^3^ iES Landau, Institute for Environmental Sciences, RPTU Kaiserslautern‐Landau Landau Germany; ^4^ Department of Biology and Environmental Science Linnaeus University Kalmar Sweden

**Keywords:** aquatic hyphomycetes, community composition, land use, leaf decomposition, organic matter, stressors

## Abstract

Fungal communities are critical for leaf decomposition, a central ecosystem function in streams. A wide range of anthropogenic stressors can alter their structure and function (i.e., leaf decomposition). Additionally, fungal communities are subject to seasonal turnover due to natural processes. Despite this, seasonality in interaction with varying stressor exposure has rarely been studied in the context of leaf decomposition. We investigated fungal community composition and leaf decomposition over one agricultural growing season by deploying leaf bags at least impacted forest and viticultural sites of 10 streams. Additionally, we transplanted leaf bags that had been colonised at the forest sites to viticultural sites to investigate how changes in stressor exposure affect the structure and function of fungal communities. Leaf decomposition was repeatedly lower in the viticultural treatment than in the forest treatment, which was partly explained by the environmental variables. The decomposition of the transplanted leaves varied across the time points and was overall more similar to that of the forest treatment. The fungal communities in April were similar across treatments, whereas all exhibited different seasonal community turnover. At later time points (June, August and September), the fungal communities from the forest and transplant treatment remained similar, likely triggered by the priority effects of the location of colonisation (forest). The viticultural treatment, however, deviated at these time points, which coincided with the timing of fungicide application. Overall, we show that both community composition and function of leaf decomposition exhibit seasonal and stressor‐related variability. Thus, our study demonstrates that seasonality and the actual stressor regime need to be considered and well described when investigating land use effects on leaf decomposition and associated fungal communities.

## Graphical abstract

Fungal communities are critical for leaf decomposition, a central ecosystem function in streams. A wide range of anthropogenic stressors can alter their community composition and decomposition. Though fungi are subject to seasonal turnover this is in interaction with varying stressor exposure rarely studied. We investigated fungal community composition and leaf decomposition over one agricultural growing season by deploying leaf bags at least impacted forest and viticultural sites of 10 streams. Additionally, we transplanted leaf bags from forests to viticultural sites to investigate how changes in stressor exposure affect the structure and function of fungal communities.

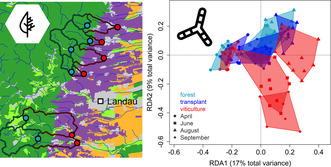

## 1 Introduction

Leaf decomposition is an important ecosystem function in streams (Graça and Canhoto 2006; Vannote et al. 1980). Allochthonous organic matter such as leaves is initially colonised by bacterial and fungal communities, which provide a high‐quality food source for macroinvertebrate shredders (Bärlocher 1985; Graça 2001). Because most allochthonous leaf input occurs in autumn decomposition is naturally subject to seasonal changes (Duarte et al. 2016; Nikolcheva and Bärlocher 2005).

Most streams are subject to multiple stressors, often associated with agriculture, which leads to chemical exposure, nutrient increase and habitat degradation (Rasmussen et al. 2013; Schäfer et al. 2016). The effect of such stressors may vary based on their magnitude but also the exposure duration. For example, while moderate nutrient increases can stimulate decomposition (Gulis and Suberkropp 2003; Truchy et al. 2022), various chemicals, including pesticides, can decrease decomposition rates as well as fungal taxa richness and alter community composition (Duarte, Pascoal, Alves, et al. 2008; Fernández et al. 2015; Rasmussen et al. 2012). With repeated pesticide exposure, as common in agricultural streams (Spycher et al. 2018; Vormeier et al. 2023), community changes can be most pronounced, while functions such as decomposition might be maintained through acclimatisation (Schreiner et al. 2018, 2023; Wiberg‐Larsen et al. 2021). Such acclimatisations can even lead to enhanced performance (e.g., expressed as decomposition) with fungicide exposure (Feckler et al. 2018; Gardeström et al. 2016).

To date, most studies investigating the response of fungal communities and leaf decomposition to stressors, including pesticides, were either conducted in the laboratory without spatial context or were field studies restricted to a single time point. The time point at which field studies were performed was mainly either in concert with maximal stressor exposure (e.g., Schäfer et al. 2007; Schreiner et al. 2023) or natural leaf input (e.g., Fernández et al. 2015; Voß et al. 2015). Similarly, community acclimatisations like the potential development of tolerance to repeated stress exposure in fungal communities have most frequently been studied under laboratory conditions (e.g., Feckler et al. 2018; Gardeström et al. 2016; Schreiner et al. 2018). Only a few studies have investigated the stress responses of fungal communities with different exposure histories, by transplanting colonised leaves (Pérez et al. 2018; Sridhar et al. 2005). To unravel patterns of community acclimatisation to repeated stressor exposure, leaf decomposition and fungal community composition need to be studied across multiple seasons and stressor conditions.

We investigated fungal community composition and leaf decomposition across the agricultural growing season of 1 year. Thereby, we were able to study potential interactions between anthropogenic stressors and environmental variables that both exhibited seasonal variations. Across 10 streams, we selected two sites: one in the least impacted, forested area and one adjacent to viticulture, exposed to multiple stressors, including fungicide exposure (Fernández et al. 2014). We compared fungal community composition via morphological identification of spores and metabarcoding as well as leaf decomposition across these two stream sites. Furthermore, a third set of leaf bags was colonised in the least impacted sites and transplanted to the viticultural sites (transplant treatment) to investigate how a least impacted community responds to different local environmental and stressor conditions. This process was repeated at four time points to capture the time before (April), during (June and August) and after (September) fungicide application in viticulture. We hypothesised that (I) decomposition in the viticultural treatment would always be lower than in the forest treatment, while (II) the decomposition of the transplant treatment would differ across seasons in comparison to the other treatments, due to differences in stressor exposure between the two stream sites. Specifically, we expected higher decomposition of the transplant treatment in comparison to the other treatments in April due to increased nutrient levels at the viticultural sites, while we expected a decreased decomposition at the later time points due to stressors such as pesticide exposure. Furthermore, we expected (III a) similar fungal communities in the forest and the transplant treatments, which were colonised in the forest, due to priority effects (Debray et al. 2021), while (III b) the fungal communities of the viticultural treatment would differ significantly, particularly during and after the fungicide application period.

## 2 Materials and Methods

### 2.1 Study Design

We conducted a field experiment in 10 streams in southwest Germany, flowing parallel from east to west with a 50 km distance between the most northern and southern streams (Figure S1). All streams are located in the same geogenic background (sandstone) and were exposed to similar weather conditions as well as land‐use changes, making them suitable replicates within field studies. At each stream, two sites with a mean distance of 5.5 ± 3.9 km (Table S1) were selected: a least impacted upstream area surrounded by forest and a downstream area with intensive viticulture exposed to multiple stressors, including fungicides. The upstream location of the least impacted sites was selected because of the absence of adjacent pesticide application areas. Despite this, pesticides were detected in non‐neglectable levels (see Section 3.1), matching observations from other years of the same study area (Schneeweiss et al. 2022) but also different least impacted sites (Wolfram et al. 2023).

We deployed leaf bags at the two stream sites to represent two treatments (termed forest and viticultural treatment). A systematic difference due to catchment area should be negligible, as a previous large‐scale study observed no relationship between decomposition and catchment area (Schreiner et al. 2023). In a third treatment, leaf bags were colonised by microbial communities at the least impacted site for 1 week and subsequently transplanted to the downstream stream section (termed transplant treatment, Table S2). This process was repeated at four time points throughout the 2018 growth period. The time points were chosen to capture the time before (April), during (June and August) and after the fungicide application (September, deployment times Table S2, reflected in the fungicide exposure Figure S2). Due to a severe drought in 2018 (Ministerium für Klimaschutz, Umwelt, Energie und Mobilität, Rheinland‐Pfalz 2023), only 6 of the 10 streams had sufficient water levels to be included in September (Table S2). During all deployment periods, the water temperatures at all 20 study sites were measured hourly using HOBO Pendant loggers (Synotech).

Furthermore, nutrient and oxygen concentrations, as well as pH and conductivity were measured every 3 weeks throughout the growing season (i.e., once per deployment period) using a compact‐photometer PF‐12 equipped with visocolor colorimetric tests (Macherey–Nagel) and a Multi 340i (WTW) (Figure S2; Ohler et al. 2023). At the same time, water samples were collected (in the scope of the Germany‐wide monitoring campaign Kleingewässermonitoring; Liess et al. 2021). Data for 76 pesticides analysed in these samples were taken from Halbach et al. (2021). The potential toxicity towards fungi was estimated by calculating the logarithmic sum of toxic units (sumTU) for the 24 detected fungicides as follows:

$$\text{sumTU}=\log\left(\sum \frac{c_{i}}{{\mathrm{EC}}_{50_{i}}}\right)$$

where *c* is the concentration of fungicide *i* and EC_50*i*_ is the concentration of fungicide *i* at which 50% of the test organisms were affected. Since EC_50_ for fungi are lacking and we anticipated that fungi react most sensitively to fungicides (Ittner et al. 2018), we selected the respectively lowest EC_50_ value for either the most sensitive freshwater algae or invertebrates. In previous studies, this allowed the establishment of relationships between the potential toxicity of fungicides and structural as well as functional aspects of fungal communities (e.g., Fernández et al. 2015). The EC_50_ values were collected using the R package Standartox (Scharmüller et al. 2020) while missing values were complemented by values from Lewis et al. (2016).

### 2.2 Assessment of Leaf Decomposition

Black alder leaves ( *Alnus glutinosa* (L.) Gaertn.) were collected in autumn 2017 shortly before senescence in a biosphere reserve close to the study area (at 49.24 N, 7.89 E). Subsequently, the leaves were quality sorted (i.e., those with mechanical damage including herbivory or infections were discarded), and petioles were removed. Leaves were then air‐dried and stored at room temperature until use. Seven replicates of leaf bags with a mesh size of 500 μm were filled with 7 ± 0.05 g of leaves for the time points of April, August and September and with 4 ± 0.05 g of leaves for the time point of June (following the protocol of a companion study, Liess et al. 2022). Seven and 14 leaf bags were deployed for approx. 3 weeks at the viticultural and forested sites, respectively, except for the September time point, where bags were deployed for 2 weeks (following the protocol of a companion study, Table S2). The shorter deployment time of the September time point likely caused a slightly higher calculated decomposition since it was normalised for the deployment time as well as the average temperature (see below) and the initial leaching of leaves is proportionally more relevant with shorter deployment periods. We assume that the effects on fungal community composition are minimal, as the sporulation rates were similar across all time points in this study (Figure S3), despite deployment time variations of 2 (September) and 3 weeks (April, June and August). This is consistent with some previous findings (Chamier 1992; Pascoal and Cássio 2004), though it contradicts other observations (Ferreira et al. 2006).

After 1 week (Table S2), seven forest leaf bags (half of those deployed) were randomly selected and transported while submerged in stream water to the viticultural site of the same stream and retrieved jointly with the viticultural leaf bags. A full set of replicates (i.e., 7) was prepared per time point and transported to and from the field to account for handling losses.

Leaf bags were gently washed in the stream weekly to remove accumulated sediment. At the end of the deployment period, the leaf bags were opened and processed on‐site by cutting eight 1‐cm‐diameter leaf disks from randomly selected leaves to identify fungal communities morphologically and via metabarcoding (details see Section 2.3). The remaining leaf mass was transported to the lab cool (approx. 4°C) and subsequently stored at −20°C. The final leaf mass was determined as the ash‐free dry mass (AFDM) to eliminate the effects of the inorganic substrate on the leaf material. The AFDM of the remaining leaf mass was determined by first oven‐drying at 60°C for 2 days, weighing to the nearest 0.01 mg, burning to ash at 500°C for 5 h, and re‐weighing after cooling to room temperature in a desiccator. The decomposed leaf mass (*DLM*, in %) was calculated per degree day to account for the study site temperature as follows:

$$\mathrm{DLM}=\frac{\left(\frac{{\text{AFDM}}_{t0}-{\text{AFDM}}_{t1}}{{\text{AFDM}}_{t0}}\right)\cdot 100\%}{T\cdot n}$$

where the AFDM values before (*t*0, normalised for handling losses) or after deployment (*t*1) are used, *T* is the mean temperature and *n* is the number of days of deployment (together summed up as degree days). The mean temperature of the transplant treatment was determined by calculating a weighted mean based on the deployment time at the respective two study sites. Additionally, the decomposition rate *k* per degree day was calculated using the same variables (David et al. 2024; Fernández et al. 2015).

### 2.3 Identification of Fungal Communities

The hyphomycete communities were identified using morphological identification of the spores (i.e., conidia) and ITS metabarcoding. Conidia sporulation was induced by orbitally shaking five leaf disks submerged in stream water from the respective collection site at 120 rpm in darkness at 16°C for 106 h. After the samples were fixed with formaldehyde (final concentration 2%), and agglomeration of conidia was prevented using 0.5% Tween80, they were stored at 4°C. From all replicates of one treatment‐stream‐time point combination, a pooled sample was created. To identify conidia, aliquots of these pooled samples were filtered through a gridded membrane filter (0.45 μm, S‐Pak‐filters, Merck) and stained with lactic acid cotton blue solution. The number of identified conidia (100 − 400× magnification) using the keys by Gulis et al. (2009) and Ingold (1942) was normalised to (1) the ratio of investigated filter surface, (2) the number and volume of single samples used to create the pooled sample and (3) the AFDM weight of the respective set of leaf disks, resulting in a corrected number of conidia per g of AFDM:

$${\text{conidia}}_{\text{corrected}}=\frac{{\text{conidia}}_{i}}{\text{AFDM}\frac{{\mathrm{vol}}_{\text{filtered}}}{{\mathrm{vol}}_{\text{total}}}\frac{A}{A_{\text{total}}}}$$

where conidia is the number of conidia counted per taxon *i*, AFDM is the total AFDM of all disks used to create the pooled sample, vol_filtered_ is the volume filtered, vol_total_ is the total volume of the pooled sample, *A* is the area of the filter examined and *A*_total_ is the total area of the filter.

The ITS metabarcoding followed the approach of Salis et al. (2023). Briefly, DNA was extracted from the pooled samples using the Qiagen DNeasy PowerPlant Pro Kit, two PCR steps were performed (first PCR with forward primer fITS7 [5′ GTG ARTCATCGAATCTTTG 3′, Ihrmark et al. 2012]) and reverse primer ITS4 (5′ TCC TCCGCTTATTGATATGC 3′, White et al. 1990), second PCR with Illumina TruSeq and Nextera PCR primers, and sequencing was conducted on two 2 × 250 bp MiSeq V2 runs at CeGaT GmbH (Tübingen, Germany). The ITS amplicon data were analysed using the DADA2 version 1.14.1 following the ITS pipeline workflow (1.8) (Callahan et al. 2016): primers were removed, sequences were filtered using standard parameters (maxEE = 2, truncQ = 2, minLen = 50, rm.phix = TRUE), samples were dereplicated, error corrected and denoised, then paired reads were merged and chimeras were removed. Subsequently, we performed taxonomic assignment using the UNITE version 02.02.2019 (dynamic classifier including global and singleton sequences of species hypotheses defined at the 97% threshold; Abarenkov et al. 2020) fungal database and calculated relative read abundances. Aquatic hyphomycetes were selected from the taxonomic list based on expert knowledge. This resulted in a total of three datasets: the whole fungal community at the amplicon sequence variants (ASV) level, the hyphomycete community at the ASV level and the hyphomycete at the species and genus level, where taxa names were extracted from the ASV data.

### 2.4 Data Analysis

To identify potential differences in leaf decomposition between the four time points and treatments, we fitted a linear mixed model (LMM) using the lme4 package (Bates et al. 2023). The decomposed leaf mass in percent was the response variable, whereas the factors ‘time point’ (categorical; April, June, August and September), ‘treatment’ (categorical; forest, transplant and vine), and their interaction were included as explanatory variables. We accounted for the nesting of treatments within a stream using ‘stream’ as a random factor (categorical, 10 levels). Because the seven replicated leaf bags per stream‐treatment‐time point combination were technical replicates, this term was included as a further random factor (categorical, 30 levels). The effects of explanatory variables and their interactions on leaf decomposition were analysed using type‐II ANOVA with *F*‐tests. To distinguish significant differences between treatments at each time point, pairwise *t*‐tests at each time point were run separately to assess differences between treatments using the R package emmeans (Lenth et al. 2023). The *p*‐values were adjusted for multiple comparisons using multivariate *t*‐distribution as described by Hothorn et al. (2008). The decomposition between the time points was not directly compared due to differences in the deployment time (see Section 2.2 ).

Differences in the fungal communities were analysed separately for morphologically identified hyphomycetes, hyphomycete taxa identified via metabarcoding, the ASVs of hyphomycetes and all fungal ASVs using redundancy analysis (RDAs; R package vegan, Oksanen et al. 2022) with Hellinger transformation to standardise the data and thereby avoid problems associated with the use of Euclidean distance for ecological data (Legendre and Gallagher 2001). Each model was fitted using the explanatory variables ‘time point’, ‘treatment’ and their interaction, which were nested in ‘streams’. The significance of the single and interacting explanatory variables was calculated using an ANOVA‐like permutation test for constrained correspondence analysis with 999 permutations (Legendre et al. 2011). Since the four different datasets on the fungal communities provided similar results, which match the results of a previous study (Salis et al. 2023), we only present the results for the morphological analysis in the main text and refer readers to the Supporting Information for metabarcoding results on the whole fungal and hyphomycete communities.

To further scrutinise changes in the fungal community, turnover and nestedness of the communities were calculated using communities from the forest treatment in April as a reference. As proposed by Baselga (2010), turnover was calculated as the difference between total dissimilarity of the beta diversity using the Simpson dissimilarity, and nestedness was estimated as the nestedness‐resultant fraction of the Sørensen dissimilarity both based on the Sorensen index using the betapart package (Baselga and Orme 2012).

Single hyphomycete taxa and ASVs were identified as indicator taxa for the explanatory variables ‘treatment’ and ‘time point’ using an Indicator Value analysis (IndVal, De Cáceres et al. 2010). Differences in taxa or ASV richness (for in total four datasets: hyphomycete taxa morphologically identified, hyphomycete taxa via metabarcoding, ASVs of hyphomycete and all fungi) across time points and treatments were analysed using a generalised linear mixed model (GLMM) with Poisson distribution following the approach of leaf decomposition analysis. If the interaction of the explanatory variables ‘treatment’ and ‘time point’ was not significant in the models (GLMM, ANOVA‐like permutation test), it was removed from the respective models.

Data preparation, statistical analysis, and graphical presentations were conducted in R (version 4.4.2, R Core Team 2024 using several additional packages, a full list of all packages used and their versions are provided in the R code). We provide all raw data and computer code under https://doi.org/10.5281/zenodo.14772677 (Schreiner et al. 2025) or https://github.com/VCSchr/Timing_matters. The raw data of the metabarcoding analysis in this study have been uploaded to the NCBI Sequence Read Archive (SRA) under BioProject accession number PRJNA1066611.

## 3 Results

### 3.1 Differences in Environmental Variables Between Stream Sections

Environmental variables differed between the forested and viticultural sites, and some also varied across seasons (Figure S2). Generally, higher water temperature, conductivity, pH, nutrient concentrations and pesticide toxicity towards fungi, as well as lower dissolved oxygen concentrations, were detected at the downstream viticultural sites. The dissolved oxygen and pH decreased over time at both site types, with lower differences in dissolved oxygen between site types during April and June in contrast to August and September.

In April and June, no fungicides were detected in forested sites, whereas the potential toxicity towards fungi at viticultural sites was low, with sumTUs of −3.6 (± 1.3, April) and −2.9 (± 0.7, June). In August and September, however, pesticide toxicity was clearly higher at both sites, with a maximum of sumTU −1.8 (± 0.4) at the viticultural sites in August. The concentrations of nitrite, nitrate and ammonium were highest at the viticultural sites in April, whereas the phosphate concentrations were highest at the viticultural sites in September.

### 3.2 Differences in Leaf Decomposition Over Time Among Treatments

**FIGURE 1:**
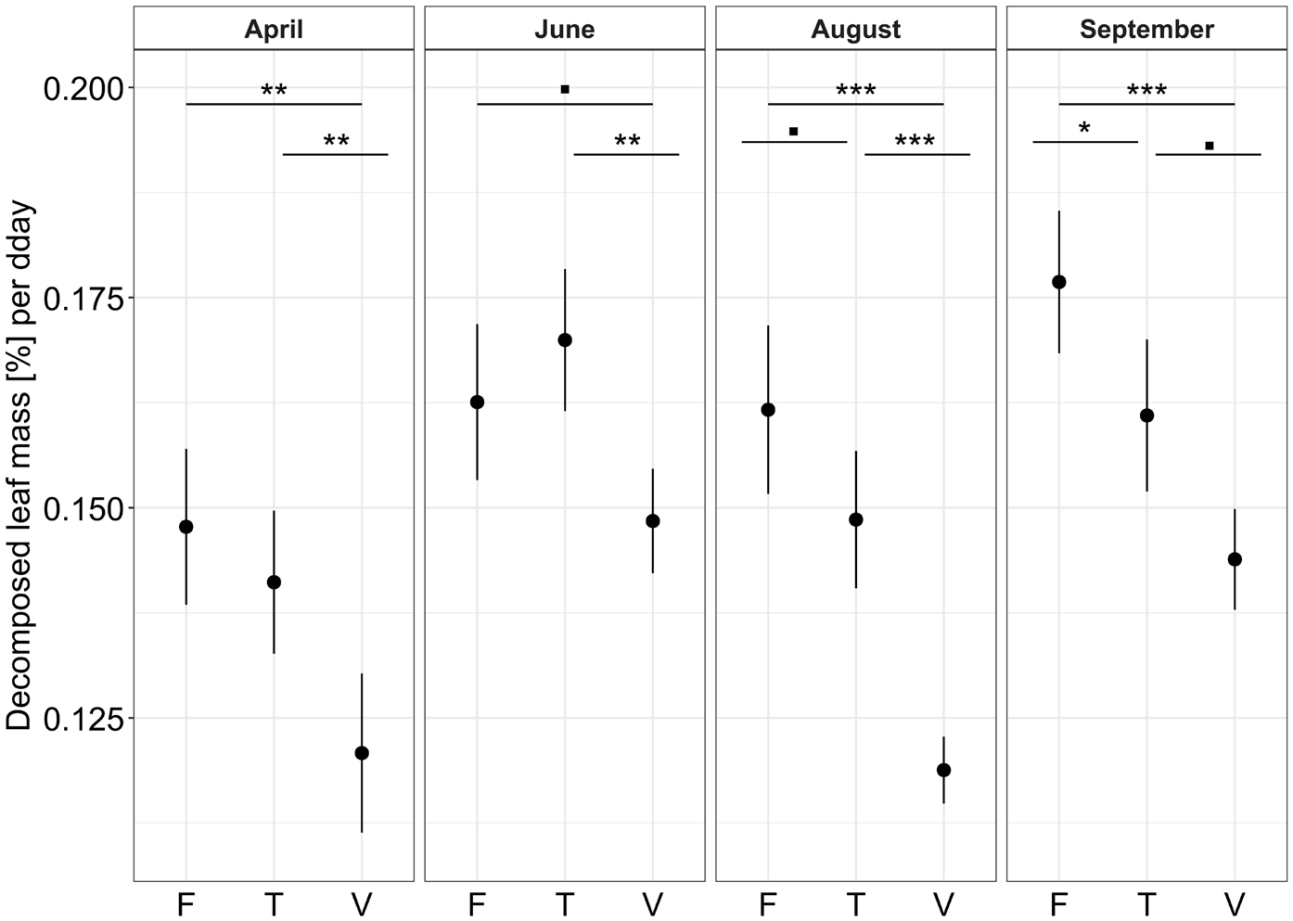
Decomposed leaf mass of the three treatments: Forest (F), transplant (T), and viticultural (V) across the four study time points (April, June, August and September) in percent per degree day (dday). The mean percentage values are presented with 95% confidence intervals. The symbols on top refer to the differences between the treatments at each time point, with ■ ≙ 0.1 > *p* > 0.05, * ≙ 0.05 > *p* > 0.01, ** ≙ 0.01 > *p* > 0.001, and *** ≙ *p* < 0.001. Exact *p*‐values are given in Table S3. During the September time point, leaf bags were only deployed at six of the 10 streams due to droughts. Given a shorter deployment time in September (see Table S2), which may influence decomposition and community composition, we abstained from comparisons across time points. A similar figure with decomposition rates *k* can be found in Figure S4.

Decomposition was consistently lower in the viticultural treatment than in the forest (Figure 1). This difference was significant at all time points (pairwise *t*‐test, *p* ≤ 0.002, Table S3), except for the June time point when the decomposition in the viticultural treatment was only slightly lower (pairwise *t*‐test, *p* = 0.057, Figure 1). The decomposition of the transplant treatment differed across time points compared to the other treatments. While the decomposition of transplant and forest treatment were initially similar (i.e., in April and June, Figure 1, Table S3), they differed slightly in August (pairwise *t*‐test, *p* = 0.075) and significantly in September (pairwise *t*‐test, *p* = 0.041). The decomposition of the transplant and viticultural treatments differed throughout the year, with the largest difference in August (pairwise *t*‐test, *p* < 0.001) and the smallest difference in September (pairwise *t*‐test, *p* = 0.051, Figure 1, Table S3).

### 3.3 Differences in Fungal Communities Across Treatments and Time

**FIGURE 2:**
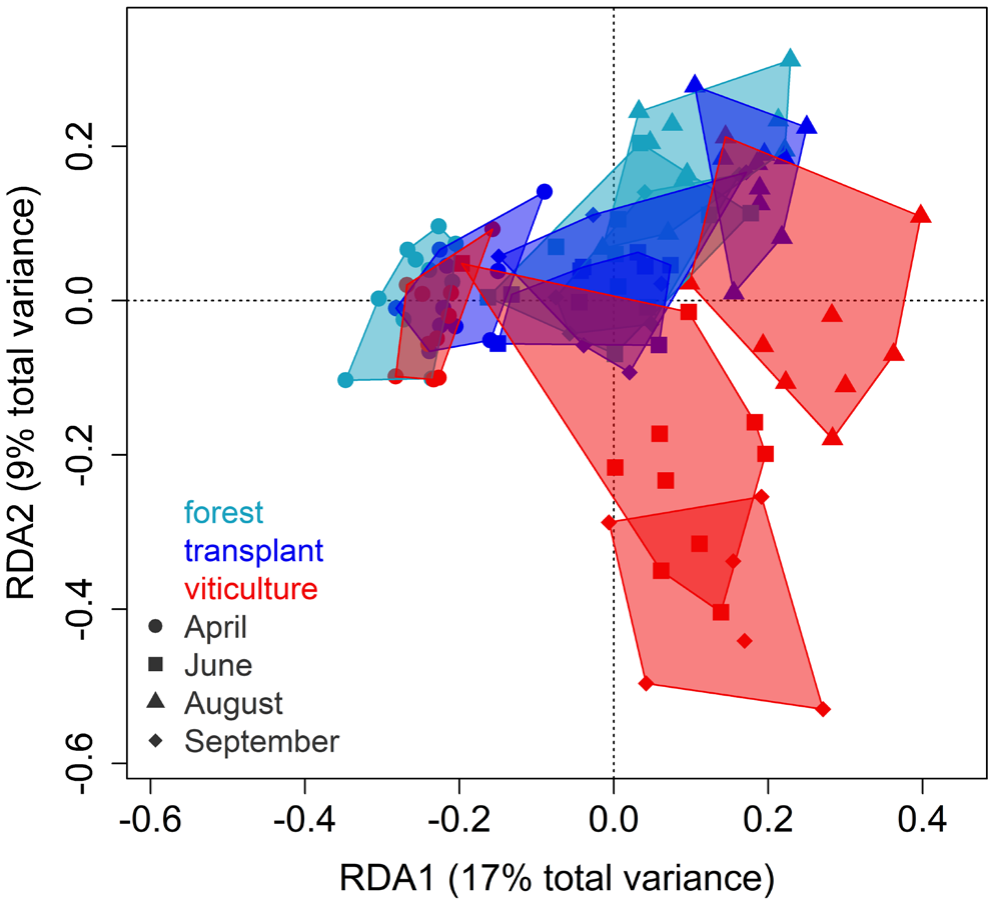
Morphologically identified hyphomycete communities across different treatments and time points. Each point within the redundancy analysis (RDA) represents the community of a single stream‐treatment‐time point combination (technical replicates were pooled before identification). The colours represent the treatments, and the shape represents the time point. The *p*‐values are provided in Table S5. During the September time point, leaf bags were only deployed at six of the otherwise 10 streams due to droughts and for a deployment time of 2 instead of 3 weeks (Table S2). The RDAs of the single time points are presented in Figure S7. A version of this figure without polygons is provided in Figure S8. The RDAs of the whole fungal community and hyphomycetes as well as ASV communities identified via metabarcoding are presented in Figure S9.

The morphologically identified hyphomycete communities responded significantly to an interaction of treatment and time point, in which the viticultural communities and those from the other treatments differed from June ongoing (Figure 2, Table S5). At the first time point (April), the hyphomycete communities of all treatments largely overlapped, indicating similar communities regardless of the treatment (Figure 2). This was supported by high nestedness across the fungal communities detected in April (Figure S3b), although community turnover from the forest to transplant and viticultural treatment was significant (ANOVA‐like permutation test, *p* = 0.0063 and *p* < 0.001, respectively, Figure S3a). The April communities differed from communities at later time points (Figure 2) and were generally characterised by a higher mean taxa richness (April: 17, June: 8, August: 7 and September: 10, Figure S6a). Furthermore, a high number of hyphomycetes (14 taxa) was identified as indicator taxa for the time point April and only a few taxa occurring in April also occurred at later time points (Figure S3b). At the other time points zero (June) to five (September) hyphomycetes were determined as indicator taxa (Table S6).

Across all later time points (June, August and September), the fungal communities in the forest and transplant treatment showed clear overlaps, hinting towards similar communities across the two treatments and time points (Figure 2). This is supported by the same three hyphomycete taxa identified as indicator taxa for both treatments (Table S7) and the strong nestedness of both the forest and transplant treatments in August and September (Figure S3b). The fungal communities of the viticultural treatment differed clearly from those of the other treatments and also showed only little overlap across all four time points (Figure 2). The strongest community turnovers were observed for the viticultural treatments in June and August as well as all treatments in September (Figure S3a). This difference between viticultural communities and the forest as well as transplant communities is supported by the fact that hyphomycete taxa were either identified as indicator taxa of the viticultural treatment or the forest and transplant treatment (for morphological as well as metabarcoding data), while no taxon was indicative of the viticultural and either the forest or transplant treatment (Table S7).

Since the results for the whole fungal community and metabarcoding‐based analyses generally agreed with the results for morphologically identified hyphomycetes, details are presented in the Supporting Information S1.

## 4 Discussion

We detected strong negative effects of viticultural land use on leaf decomposition across the four studied time points, which confirms Hypothesis I. The leaf decomposition of the transplant treatment varied across different time points compared to that of the forest and viticultural treatment, supporting Hypothesis II. While the fungal communities of forest and transplant treatments strongly overlapped (confirming Hypothesis IIIa), the communities of the viticultural treatment differed significantly for time points during or after pesticide application, which partially confirms Hypothesis IIIb. The results based on morphological identification and metabarcoding of the fungal communities revealed similar patterns, which matches the findings of a previous study (Salis et al. 2023).

### 4.1 Differences in Leaf Decomposition Over Time Among Treatments

As expected (see Hypothesis I), leaf decomposition was always lower in the viticultural treatment than in the forest treatment. This is in line with a wide range of studies that detected reduced decomposition in anthropogenically altered stream sections (Cabrini et al. 2013; Fernández et al. 2015; Lecerf and Chauvet 2008; Rasmussen et al. 2012; Schäfer et al. 2012; Voß et al. 2015). But contrasting studies did not detect such land use‐induced decomposition changes (Hagen et al. 2006; Piscart et al. 2009). This reduction in decomposition is likely related to a change in environmental variables, of which, in particular, temperature and conductivity differed between forested and viticultural sites (Figure S2). The difference in leaf decomposition between the forest and viticultural treatments was most pronounced in August (Figure 1), where we also detected the highest potential toxicity towards fungi (Figure S2). This is in concert with a field study conducted in the same study region, where leaf decomposition was related to a gradient of pesticide toxicity (Fernández et al. 2015). The significant differences in leaf decomposition in April, however, were likely not driven by potential toxicity. This is because the potential toxicity was below an effect threshold (log sum toxic unit of −3) that was associated with minor effects in earlier studies (for invertebrates: Schäfer et al. 2012; for decomposition: Rasmussen et al. 2012). The potential toxicity towards fungi may have contributed but was not the main driver in differences in leaf decomposition across treatments in spring.

The decomposition of the transplant treatment compared with the other two treatments varied across the study time points supporting Hypothesis II. We expected increased decomposition of the transplant treatment compared to the forest treatment during April due to elevated nutrient concentrations (Figure S2), which are known to stimulate decomposition at the observed levels (Gulis and Suberkropp 2003; Truchy et al. 2022). However, leaf decomposition was non‐significantly reduced in April (Figure 1), which matches a meta‐analysis (Beaumelle et al. 2020) and might be related to the influence and potential interaction of several other environmental variables besides nutrients in the field context. Generally, leaf decomposition of the transplant treatment was more similar to the decomposition of the forest than the viticultural treatment (except for the September time point, Figure 1). This is in concert with a study in which a similar decomposition was observed in bags transplanted to a polluted site compared to the non‐transplanted ones (Duarte, Pascoal, and Cássio 2008). However, other studies found an intermediate decomposition of transplant treatments between the decomposition in polluted and non‐polluted sites or the decomposition even resembled that of the polluted sites (Pérez et al. 2018; Sridhar et al. 2005). The maintenance of leaf decomposition in transplant treatments in sites with higher stress levels was attributed to a higher taxa richness in such treatments in a previous study (Duarte, Pascoal, and Cássio 2008). However, this cannot explain the similar leaf decomposition levels in our study, as taxon richness was similar in April and June across all treatments (Figure S6). Generally, it is rather the functional attributes than the taxa richness in the communities that may determine changes in leaf decomposition.

### 4.2 Differences in Fungal Communities Across Treatments and Time

As Hypothesis (IIIa), the fungal communities in the forest and transplant treatment overlapped and exhibited only minor community turnover, whereas the communities in the viticultural treatment significantly differed at most time points from communities of the forest and transplant treatments (IIIb, Figure 2). The similarity between the communities from the forest and transplant treatment can be explained by their colonisation in the same stream sections and the related priority effects of an established community (Debray et al. 2021). The overlap of communities from the two locations was demonstrated using DNA metabarcoding and morphological identification, although the latter is only able to detect taxa that can still reproduce (Wutkowska et al. 2019). Similar community overlaps between the original (i.e., non‐transplanted) and transplanted fungal communities were detected in previous studies in summer, in which established communities persisted on colonised leaves (Sridhar et al. 2005; Suberkropp 1984). By contrast, one study in winter found that the community composition and taxa richness of transplanted communities, subject to higher temperatures and nutrient levels, differed from those of their colonisation location (Pérez et al. 2018). These contrasting results suggest that the persistence of established fungal communities depends on the season. The higher activity of fungal communities during autumn and winter likely makes transplanted communities more susceptible to environmental changes, as observed in by Pérez et al. (2018). In contrast, spring and summer communities are less subjected to stress‐induced community turnover.

The similarity between the communities of the viticultural and the other two treatments in April contrasts with the reduction in leaf decomposition (see Section 4.1). This confirms that structural and functional responses are not always tightly linked (Feckler and Bundschuh 2020). Indeed, other studies detected changes in community composition without altered leaf decomposition (Baudy et al. 2021; Feckler and Bundschuh 2020; Salis et al. 2023). The deviation of fungal communities from the viticultural treatment to other treatments at later time points during and after the fungicide application (i.e., June, August and September) is in accordance with previous studies (e.g., Fernández et al. 2015; Voß et al. 2015). Also, the reduction in taxa richness (Figure S6a) in August and September due to multiple‐stressor exposure (i.e., of the viticultural treatment) is consistent with earlier field (Fernández et al. 2015) and laboratory studies (Bundschuh et al. 2011; Feckler et al. 2018), where a similar reduction was detected between exposed and non‐exposed communities or communities from reference sites. The taxa richness of viticultural communities was only reduced at later time points (i.e., August and September) but not in June when the community was already significantly changed and exposed to multiple stressors such as fungicides and higher temperatures. This suggests a low reproduction ability of stressed fungal species, which hinders their colonisation of new substrates and consequently their exclusion from the fungal communities at later time points.

Various taxa were identified as indicator taxa for the treatments. Among them, *Tetracladium* sp. was indicative of forest treatment, which is in contrast to previous studies (Bollinger et al. 2022; Bundschuh et al. 2011), where the species *Tetracladium marchalianum* occurred irrespective of treatment and maintained its decomposition ability under high fungicide concentrations in the laboratory (Baudy et al. 2021). Supporting our observations was a study that detected decreasing sporulation rates of *T. marchalianum* with increasing temperatures, as observed at viticultural sites (David et al. 2024), likely due to the lower temperature optima of *Tetracladium* species (Dang et al. 2009). Furthermore, *Lunulospora curvula* is an indicator taxon for viticultural treatment, which is in concert with studies that reported that this species is tolerant to zinc exposure and changes in water chemistry (Cudowski et al. 2015; Duarte et al. 2004; Rajashekhar and Kaveriappa 2003). In contrast to these studies, as well as our observations, Flores et al. (2014) observed a decline in *L. curvula* following exposure to fungicides. Additionally, *L. curvula* is a known warm‐adapted species that dominated in viticultural communities (Bärlocher 1991; Suberkropp 1984), due to the continuously higher temperatures at these stream sections (Table S2) The species *Tetrachaetum elegans* and *Articulospora tetracladia* were both identified as indicator taxa of forest and transplant treatments, which is in concert with studies that observed decreased occurrences of the former with increasing pesticide exposure (Fernández et al. 2015). Furthermore, the latter thrived at low nutrient levels in previous studies (Fernández et al. 2016), conditions similar to those of the forested stream sections of our study.

In addition to differences in fungal community composition between treatments, we observed clear seasonality in community composition and dominating taxa. This seasonal community turnover occurred in all sites (Figure 2). The community turnover from the spring time point of April to the later time points in Summer as June, August and September in our study, is well established (Gessner et al. 1993). A further community change with increased taxa richness is expected to be associated with the main leaf‐falling period in autumn or early winter (Gessner et al. 1993). The deviation in fungal community composition from April to later time points can be attributed to the highest observed taxa richness in April (Figure S6), although previous studies detected higher taxa richness in summer and autumn (Iberian Peninsula, Mora‐Gómez et al. 2016; Canada, Nikolcheva and Bärlocher 2005) or observed similar taxa richness across seasons (Iberian Peninsula; Duarte et al. 2016). Such diverging findings might be related to the respective study regions and climates with varying temperature regimes across seasons. Indeed, the seasonality of fungal communities is related to the various temperature optima of the fungal taxa (Bärlocher 1991, 2000; Nikolcheva and Bärlocher 2005). The strongest community shifts occur when the temperature decreases below 5°C, where cold‐adapted communities dominate, which would likely occur in winter, but was not the subject of the present study, or when the temperature exceeds 16°C–18°C, where warming‐induced community changes have been observed (Gessner et al. 1993; Suberkropp 1984). As mentioned above, *L. curvula*, is a warm‐adapted species that was identified in the present study as an indicator taxon for the time point of August (and the viticultural treatment, see above). Also, other studies reported the highest occurrence of this species in summer (Bärlocher 1991; Suberkropp 1984).

Our results hint that fungal community turnover including a significantly reduced taxa richness is occurring after prolonged multiple‐stressor exposure. This is likely related to the fact that stressor exposure only slightly affects established fungal communities, but the reproduction and colonisation of new substrates. The stress‐induced changes in the fungal community were transitory in our study, as the fungal communities from the forest and viticultural sites were similar in April. This is possibly related to recolonisation from upstream refuges that occurred during winter or related to natural turnovers of fungal communities. To test the former assumption, studies without upstream recolonisation sections in different land uses are required. In the absence of such recolonisation sections, stress‐related alterations of fungal communities might be permanent and lead to an irreversible loss of biodiversity and related ecosystem functions like leaf decomposition, and finally ecosystem services.

## 5 Conclusion

Our study suggests that the temporal dynamics of fungal communities and associated leaf decomposition are related to natural seasonality but also dynamics of stressors associated with agriculture, such as pesticide exposure, elevated nutrients and increased temperature. Attributing the dynamics to a single stressor was not possible, likely due to many co‐occurring and potentially interacting stressors. Land use‐related alterations in the structure of the fungal community and its functioning, including energy provisioning in particular in small streams may have cascading effects on whole stream food webs. Our results demonstrate that future studies not only on fungal communities and leaf decomposition but also various organism groups and ecosystem functions should consider seasonal community dynamics and varying stressor exposure. This approach ensures gaining knowledge on real‐world stress responses and processes, which are required to enable reliable predictions. This knowledge, together with a detailed description of the actual stressor regime, is ultimately necessary to manage ecosystems and protect biodiversity.

## Author Contributions

**Verena C. Schreiner:** conceptualization, formal analysis, investigation, methodology, visualization, writing – original draft, writing – review and editing. **Moritz Link:** investigation, writing – review and editing. **Gesa Amelung:** investigation, writing – review and editing. **Katharina Ohler:** investigation, writing – review and editing. **Romana Salis:** investigation, writing – review and editing. **Florian Leese:** funding acquisition, writing – review and editing. **Ralf B. Schäfer:** conceptualization, funding acquisition, writing – review and editing.

## Conflicts of Interest

The authors declare no conflicts of interest.

## Supporting information


Data S1.

## Data Availability

The data and the code for all statistical analyses and figures that support the findings of this study are openly available in Zenodo at https://doi.org/10.5281/zenodo.14772677 and Github at https://github.com/VCSchr/Timing_matters. Metabarcoding analysis data are available in the NCBI Sequence Read Archive (SRA) under BioProject accession number PRJNA1066611.
