# Supplementary material for: Timing Matters: Viticultural Land Use Determines Responses in Structure and Function of Fungal Stream Communities Across One Growing Season
Source: Glob Chang Biol. 2025 Feb 13;31(2):e70085. doi: 10.1111/gcb.70085 (PMC11822880; doi:10.1111/gcb.70085)
Supplement: Supplementary file 1 — Data S1. [file GCB-31-e70085-s001.pdf]

Supporting information for

Timing matters: Viticultural land use determines responses in structure and function of fungal stream communities across one growing season

Verena C. Schreiner[1,2,*], Moritz Link[3], Gesa Amelung[3], Katharina Ohler[3], Romana Salis[1,4], Florian Leese[1] & Ralf B. Schäfer[1,2]

[1] Faculty of Biology, University of Duisburg-Essen, Universitätsstrasse 2, 45141 Essen, Germany

[2] Research Center One Health Ruhr, University Alliance Ruhr, Universitätsstrasse 2, 45141 Essen, Germany.

[3] iES Landau, Institute for Environmental Sciences, RPTU Kaiserslautern-Landau, 76829 Landau, Germany

[4] Department of Biology and Environmental Science, Linnaeus University, 39231 Kalmar, Sweden

Present address:

Gesa Amelung, Federal Environmental Agency UBA, 06844 Dessau-Roßlau, Germany

Correspondence: verena.schreiner@uni-due.de

This document contains supporting information for the manuscript titled above and adds nine figures, seven tables and one text over 17 pages.

All raw data and the corresponding computer code can be found under https://github.com/VCSchr/Timing_matters.

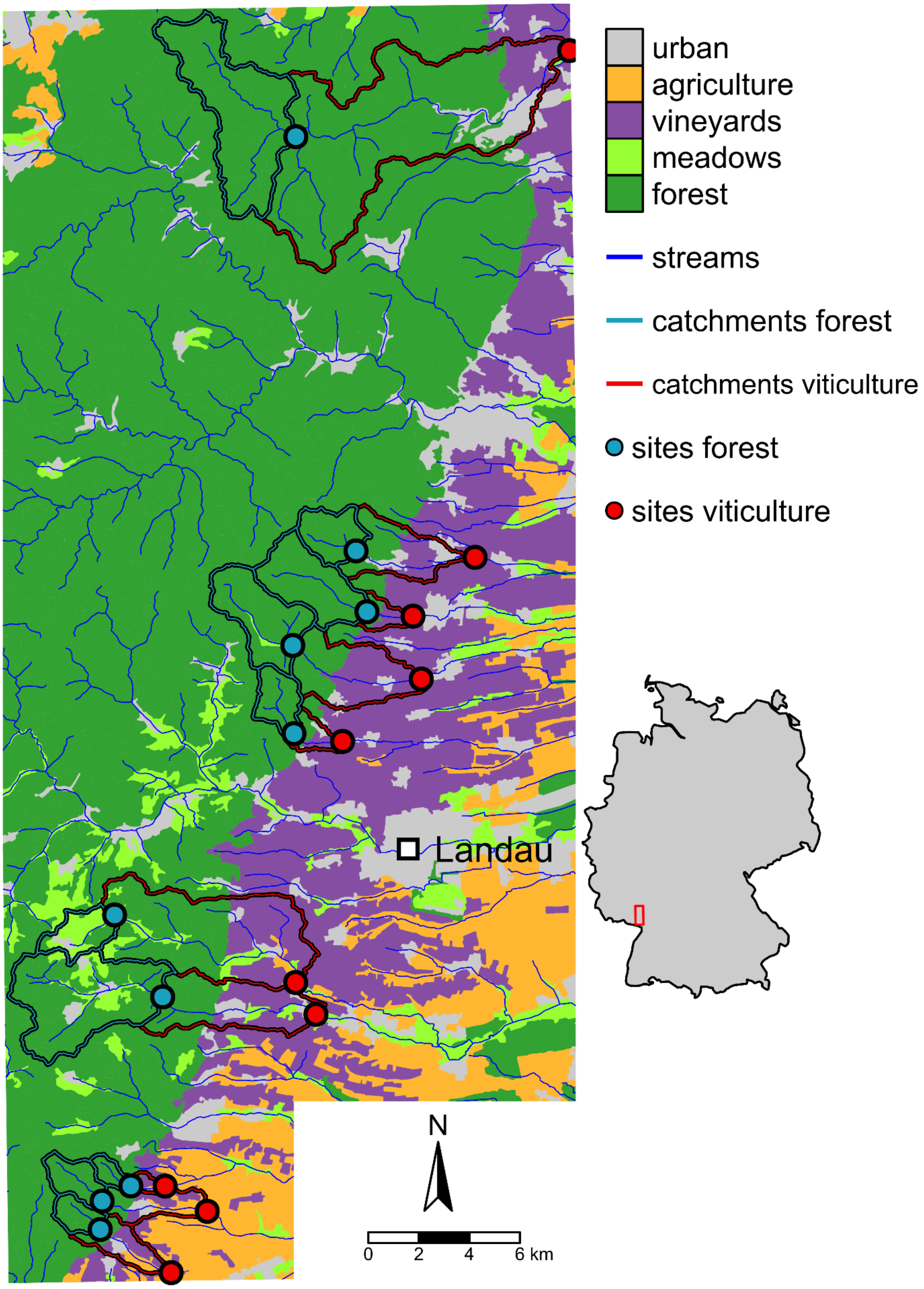

Figure S1: Sampling sites located in the ten parallel flowing streams. The inset map shows the location of the sampling sites within Germany. The map was created using the R package tmap (Tennekes et al., 2022). Land use is based on CORINE Land Cover (European environmental agency, 2019). Urban land use refers to type 1, Artificial surfaces. Agricultural land use refers to types 211, non-irrigated arable land, 222, fruit trees and berry plantations and 242 complex cultivation patterns. Vineyards refers to type 221. The map lines delineate our study area and do not necessarily depict accepted national boundaries.

Table S1: Elevation over sea level of sampling sites per stream and distance between them along the stream course. Streams are sorted from north to south.

Stream	elevation [m] forest site	elevation [m] viticultural site	distance [km]
Isenach	223	109	14.0
Kropsbach	316	145	5.3
Triefenbach	224	179	1.9
Modenbach	271	165	6.1
Hainbach	298	221	2.1
Kaiserbach	262	169	9.4
Klingbach	193	154	6.8
Dierbach	262	199	1.4
Otterbach	245	185	4.7
Russbach	259	167	3.4

Table S2: Leaf bag deployment, transplantation and retrieval dates and duration for the four time points.

Time point	Deployment date	Transplantation date	Retrieval date	Deployment duration [days]
April	19./20.03.2018[a]	26./27.03.2018	11./12.04.2018	23
June	16./17.05.2018	23./24.05.2018	05./06.06.2018	20
August	10./11.07.2018	18./19.07.2018	01./02.08.2018	22
September[b]	21./22.08.2018	28./29.08.2018	03./04.09.2018	14

[a] The first day always refers to the actions at the streams Isenach, Kropsbach, Triefenbach, Modenbach, and Hainbach (north of Landau), while the second day refers to actions at the streams Kaiserbach, Klingbach, Dierbach, Otterbach, and Russbach (south of Landau, Figure S1).

[b] During the September deployment leaf bags were only deployed at six of the ten study streams (Isenach, Triefenbach, Modenbach, Kaiserbach, Klingbach, and Otterbach) due to a severe drought at the remaining streams.

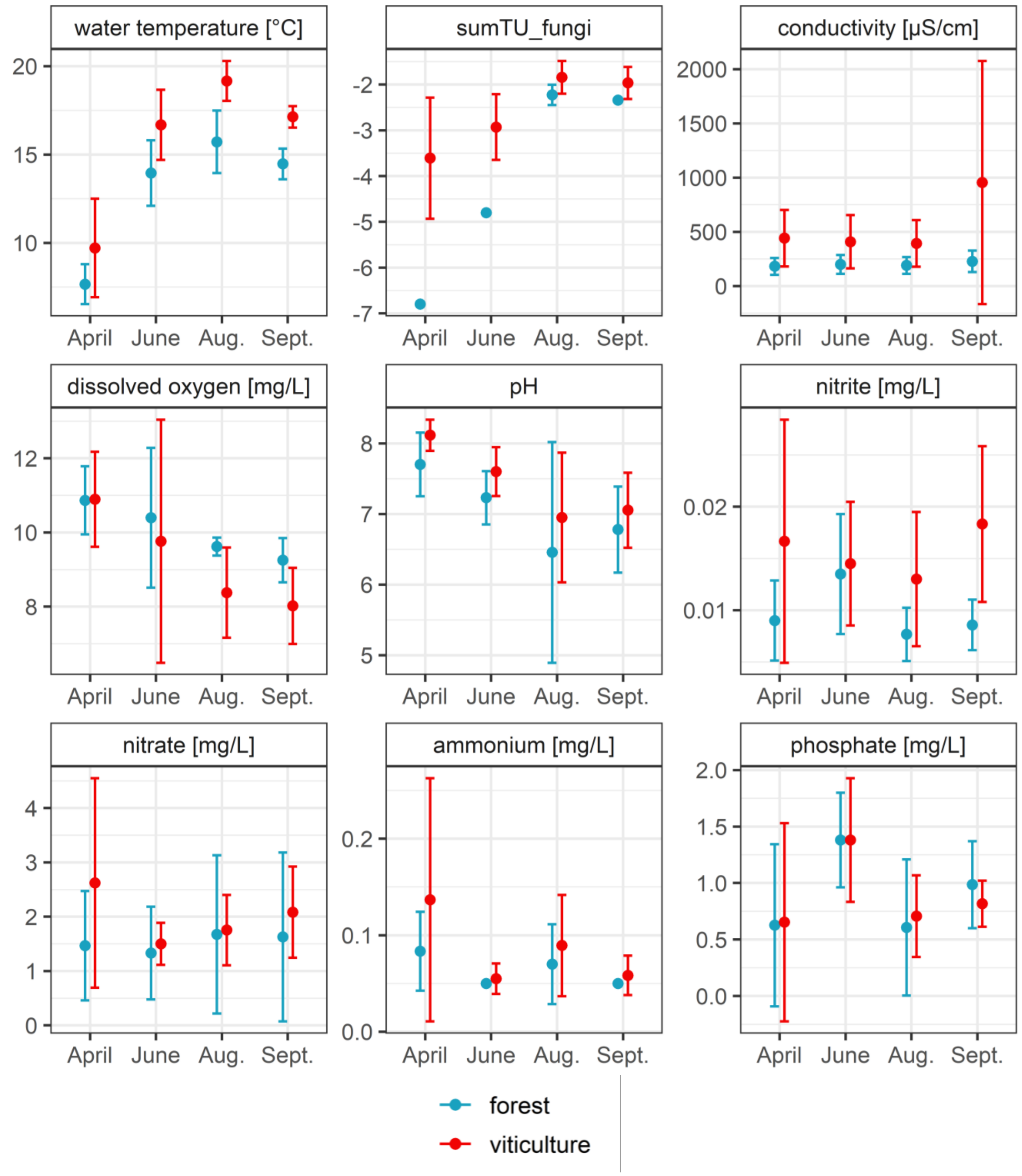

Figure S2: Mean values of environmental variables across the stream sites in forest and viticulture and the four study time points (April, June, August, September; with 95 % confidence interval). SumTU-fungi refers to the potential toxicity of fungicides towards fungi (details see section “study design” of the main manuscript). In April and June, no fungicides were detected in forested sites. Here, we assigned a sumTU corresponding to 1/10th of the minimum sumTU observed at sites with measured fungicides (sumTU -6.8 in April, sumTU -4.8 in June). Values in September only refer to six of the ten streams due to droughts (see Table S2). Due to the high number of tests necessary, we refrained from formal statistical analysis.

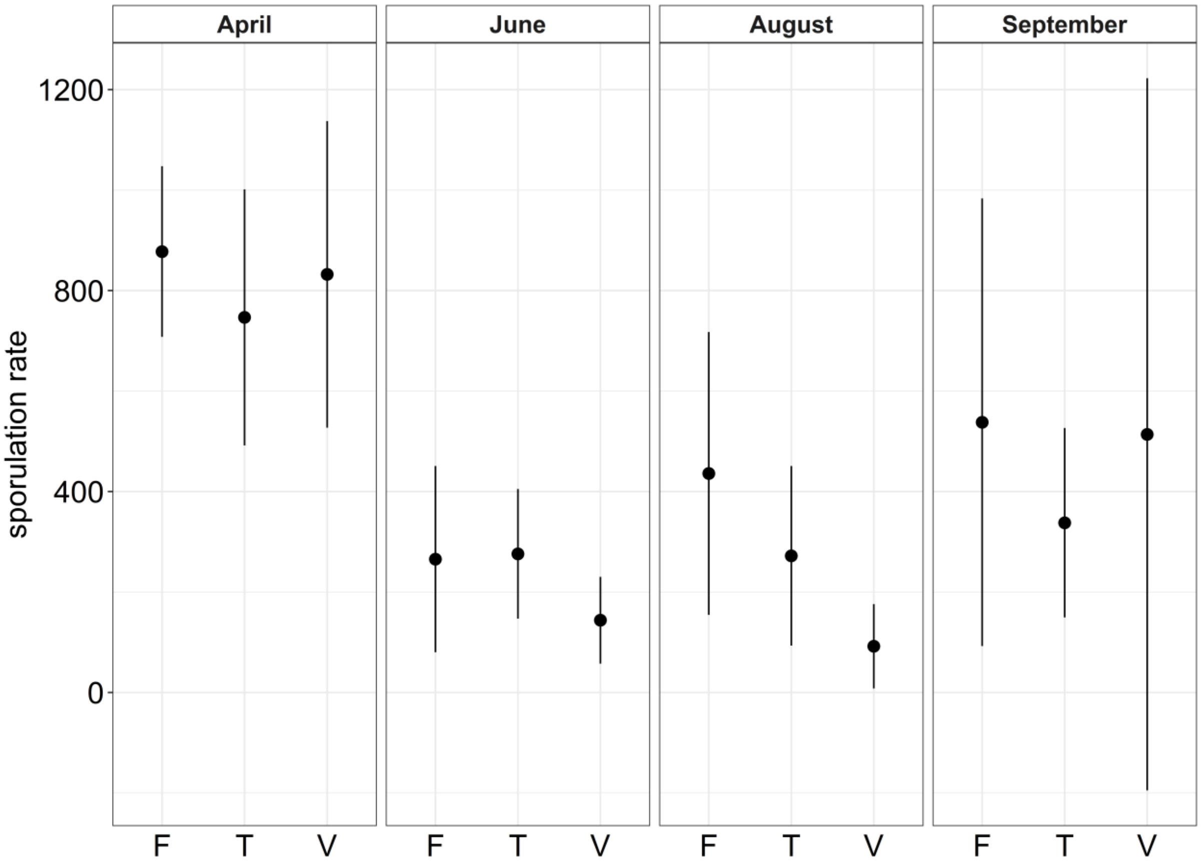

Figure S3: Sporulation rate of aquatic hyphomycetes of the three treatments: forest (F), transplant (T), and viticultural (V) across the four study time points (April, June, August and September). Mean values are given with 95 % confidence intervals. During the September time point, leaf bags were only deployed at six of the otherwise ten streams due to droughts and for a deployment time of two instead of three weeks (Table S2).

Table S3: Differences in decomposed leaf mass between treatments across the four study time points. Bold p-values indicate statistical significance. Comparisons were done using the package emmeans (Lenth et al., 2023).

Time point	Contrast between	df	t-ratio	p-value
April	forest - transplant	70.4	0.788	0.711
	forest - viticulture	69.5	4.273	**0.002**
	transplant - viticulture	73.5	3.417	**0.003**
June	forest - transplant	74.3	-1.080	0.592
	forest - viticulture	71.7	2.340	0.057
	transplant - viticulture	65.8	3.515	**0.002**
August	forest - transplant	65.7	2.220	0.075
	forest - viticulture	64.3	7.105	**< 0.001**
	transplant - viticulture	64.3	4.869	**< 0.001**
September	forest - transplant	109.4	2.456	**0.041**
	forest - viticulture	108.0	4.858	**< 0.001**
	transplant - viticulture	111.3	2.367	0.051

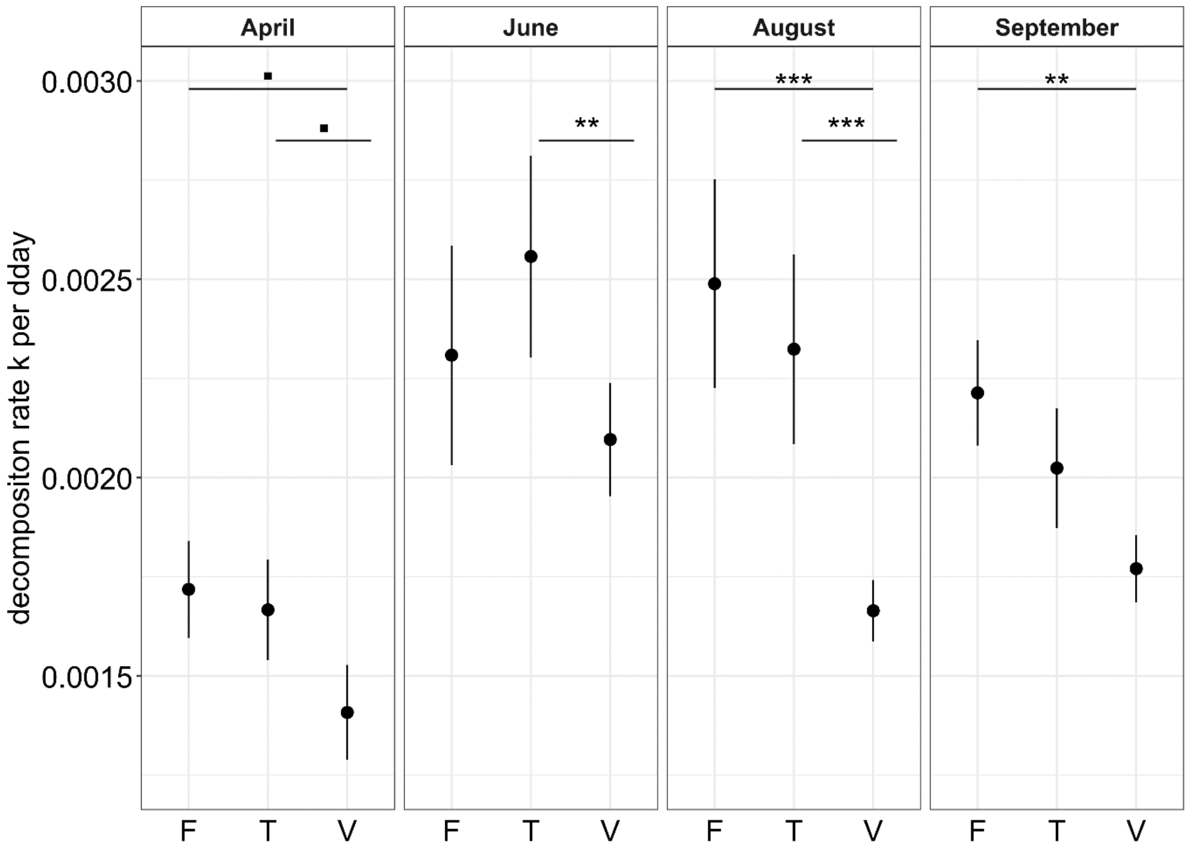

Figure S4: Decomposition rates *k* of the three treatments: forest (F), transplant (T), and viticultural (V) across the four study time points (April, June, August and September) in percent per degree day (dday). The mean percentage values are presented with 95 % confidence intervals. The symbols on top refer to the differences between the treatments at each time point, with ■ ≙ 0.1 > p-value > 0.05, * ≙ 0.05 > p-value > 0.01, ** ≙ 0.01 > p-value > 0.001, and *** ≙ p-value < 0.001. Exact p-values are given in Table S4. During the September time point, leaf bags were only deployed at six of the ten streams due to droughts. Given a shorter deployment time in September (see Table S2), which may influence decomposition and community composition, we abstained from comparisons across time points.

Table S4: Differences in decomposition rate *k* between treatments across the four study time points. Bold p-values indicate statistical significance. Comparisons were done using the package emmeans (Lenth et al., 2023).

Time point	Contrast between	df	t-ratio	p-value
April	forest - transplant	90.6	-0.099	0.995
	forest - viticulture	89.4	2.156	0.085
	transplant - viticulture	944	2.210	0.075
June	forest - transplant	96.0	-1.785	0.180
	forest - viticulture	92.4	1.683	0.217
	transplant - viticulture	84.3	3.584	**0.002**
August	forest - transplant	84.2	1.413	0.339
	forest - viticulture	82.1	6.558	**< 0.001**
	transplant - viticulture	82.2	5.132	**< 0.001**
September	forest - transplant	130.0	1.305	0.395
	forest - viticulture	128.4	3.182	**0.005**
	transplant - viticulture	132.6	1.852	0.157

Table S5: Explained variance and p-values of the single redundancy analysis (RDA) run per type of fungal community with Hellinger transformed community data and stream as a random factor. Bold p-values indicate statistical significance.

Type of Community	Explanatory variable	df	F-value	p-value	Explained variance [%]
Hyphomycetes taxa via conidia	Time point	3	11.0	**0.001**	22.7
	Treatment	2	3.96	**0.001**	6.8
	Time point x Treatment	6	2.37	**0.001**	37.7
Hyphomycetes taxa via metabarcoding	Time point	3	11.9	**0.001**	18.9
	Treatment	2	6.08	**0.001**	7.80
	Time point x Treatment	6	1.17	0.247	26.7
Hyphomycetes ASV level	Time point	3	6.36	**0.001**	11.1
	Treatment	2	5.19	**0.001**	6.5
	Time point x Treatment	6	1.16	0.170	21.2
All fungi ASV level	Time point	3	7.16	**0.001**	12.9
	Treatment	2	3.10	**0.001**	4.25
	Time point x Treatment	6	1.13	0.181	21.0

df = degrees of freedom, ASV = amplicon sequence variants

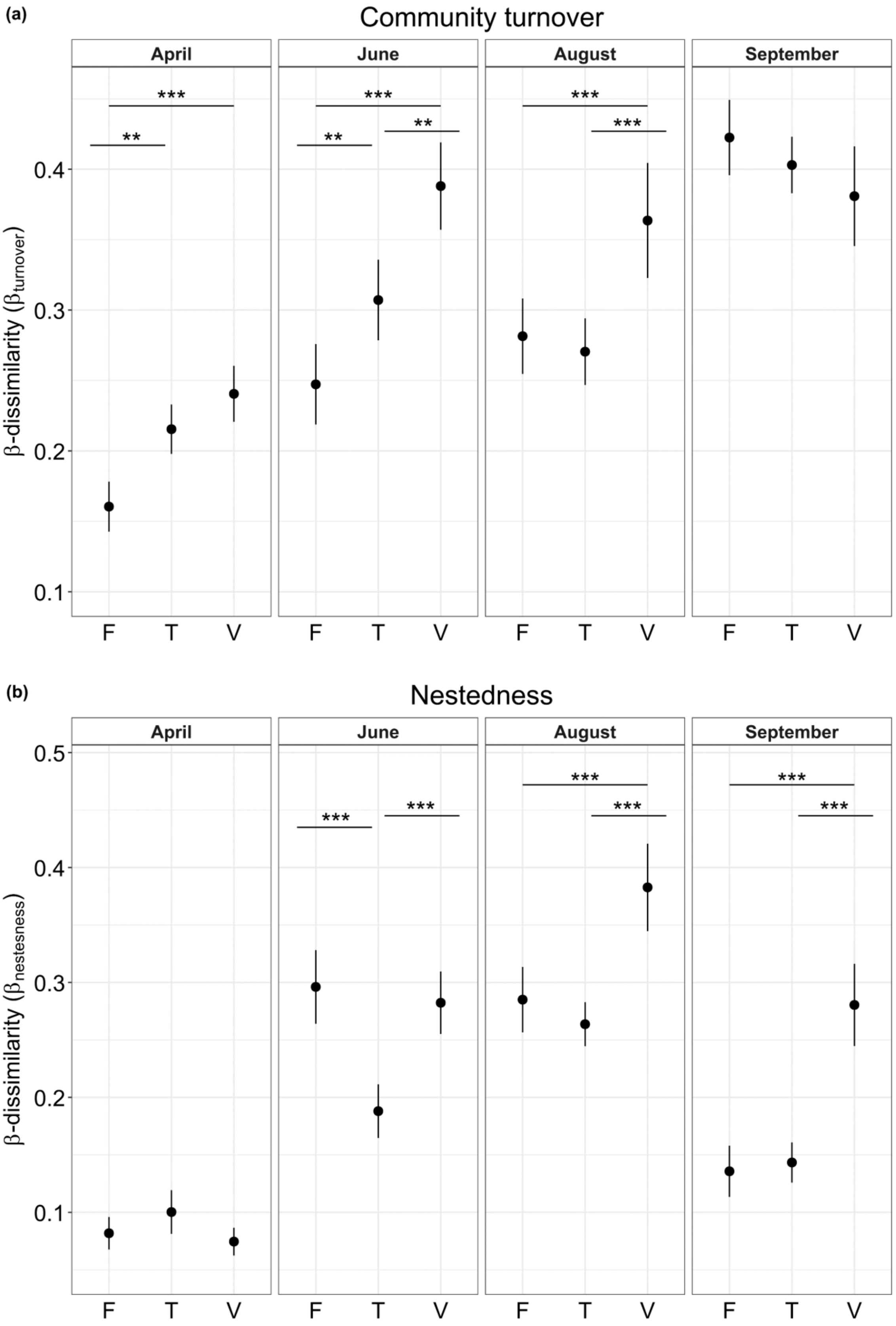

Figure S5: Community turnover (a) and nestedness (b) of the fungal communities identified morphologically calculated using the package betapart (Baselga and Orme, 2012) of the three treatments: forest (F), transplant (T), and viticultural (V) across the four study time points (April, June, August and September). The communities were compared to the ten April communities of the forest treatment. The mean values are given with 95 % confidence intervals. Symbols on top refer to the differences between the treatments at each time point, with ■ ≙ 0.1 > p-value > 0.05, * ≙ 0.05 > p-value > 0.01, ** ≙ 0.01 > p-value > 0.001.

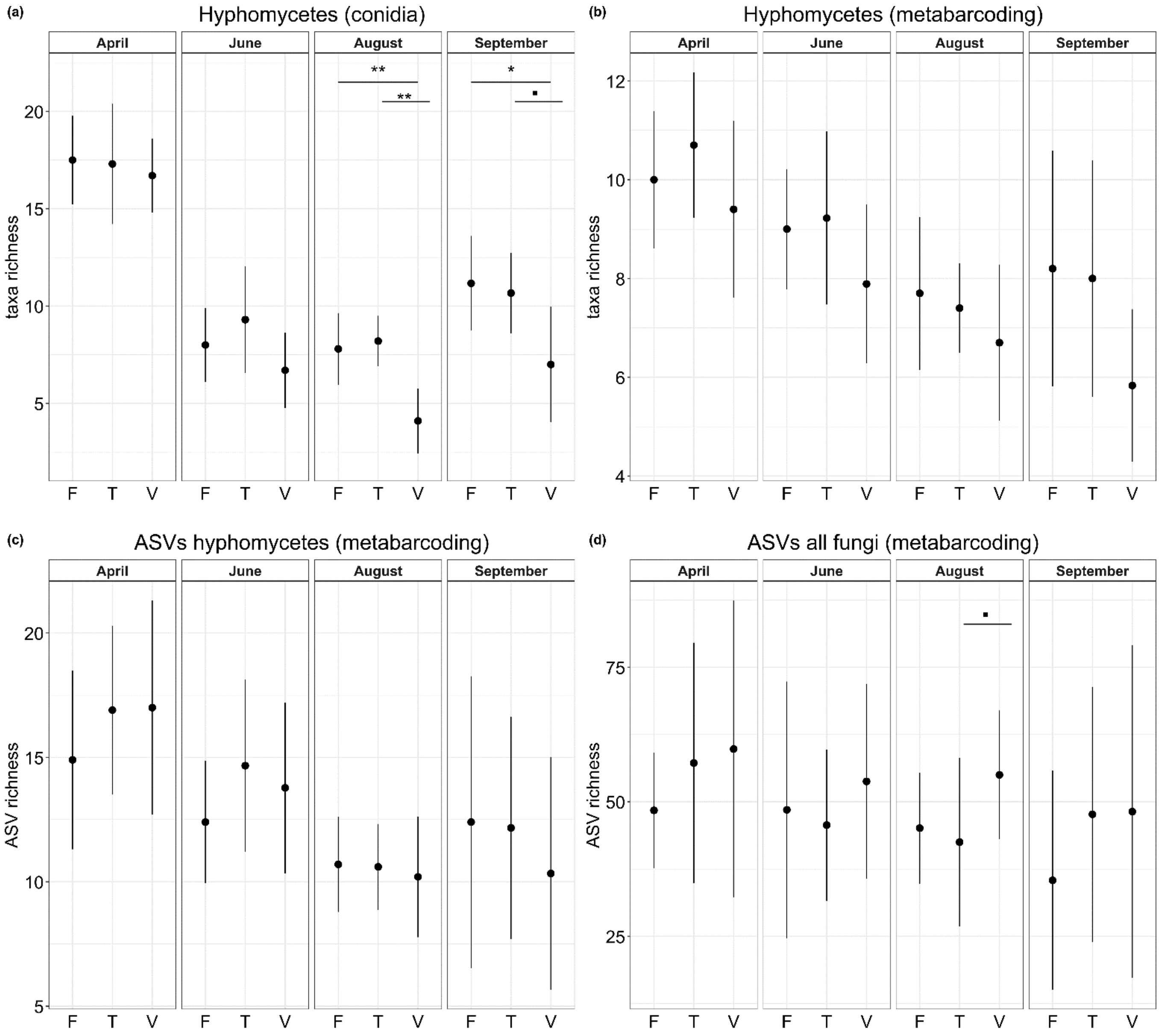

Figure S6: Taxa and amplicon sequence variants (ASV) richness of (a) hyphomycete taxa identified morphologically, (b) hyphomycete taxa identified via metabarcoding, (b) ASVs of hyphomycetes and (d) ASV of all fungi of the three treatments: forest (F), transplant (T), and viticultural (V) across the four study time points (April, June, August and September). The mean values are given with 95 % confidence intervals. Symbols on top refer to the differences between the treatments at each time point, with ■ ≙ 0.1 > p-value > 0.05, * ≙ 0.05 > p-value > 0.01, ** ≙ 0.01 > p-value > 0.001.

Table S6: Results from the Indicator Taxa Analysis (IndVal, De Cáceres et al. (2010)) for the time points of the study. A refers to the proportion that one taxon or an amplicon sequence variant (ASV) was found at a single time point. B refers to the proportion of one taxon or ASV occurring in all samples of that time point. Only taxa or ASVs that were significantly related to one or several time points are given in this table. The taxa names added to the ASVs in brackets were assigned using UNITE (Abarenkov et al., 2020) database.

Hyphomycete taxa identified with morphological identification

Time point	Taxon	A	B	IndVal	p-value
April	*Alatospora acuminata*	0.8613	1.0000	0.928	0.001
	Flagellospora fusarioides	0.8723	0.9000	0.886	0.001
	Sigmoid_A	1.0000	0.7667	0.876	0.001
	Filiform_B	0.9947	0.6333	0.794	0.001
	Heliscus lugdunensis	0.8066	0.7667	0.786	0.001
	Fontanospora eccentrica	0.9731	0.6333	0.785	0.001
	Lemoniera terrestris	0.8339	0.7333	0.782	0.001
	Articulospora tetracladia	0.7309	0.8333	0.78	0.001
	Alatospora pulchella	1	0.5333	0.73	0.001
	Lemoniera pseudofloscula	1	0.4333	0.658	0.001
	Unknown_A	0.8357	0.4667	0.624	0.001
	Unknown_B	0.8829	0.3667	0.569	0.001
	Filiform_A	1	0.1333	0.365	0.03
	Lemoniera centrosphaera	0.9554	0.1333	0.357	0.043
August	*Lunulospora curvula*	1	0.3448	0.587	0.001
	Anguillospora crassa	1	0.1034	0.322	0.044
September	*Lemoniera aquatica*	0.9158	0.7222	0.813	0.001
	Lunulospora sp.	0.9538	0.5556	0.728	0.001
	Tetracladium sp.	1	0.3333	0.577	0.001
	Heliscus tentaculus	0.8084	0.3889	0.561	0.001
	Campylospora chaetocladia	0.8901	0.2222	0.445	0.001
April+August	*Mycocentrospora angulata*	1	0.322	0.567	0.008
April+June	*Tricladium splendens*	0.9116	0.2034	0.431	0.032
April+September	*Tetracladium marchalianum*	0.8987	0.7292	0.81	0.001
	Anguillospora rosea	0.8874	0.6667	0.769	0.001
	Cylindrocarpon aquaticum	0.8996	0.2083	0.433	0.009
August+June	*Tricladium chaetocladium*	0.964	0.3621	0.591	0.003
April+August+September	*Anguillospora furtiva*	1	0.3506	0.592	0.001
April+June+September	*Anguillospora longissima*	0.9366	0.5325	0.706	0.002
	Lemoniera sp.	1	0.2857	0.535	0.005

Hyphomycete taxa identified with metabarcoding

April	*Filosporella annelidica*	0.9094	0.9333	0.921	0.001
September	*Triscelophorus monosporus*	0.9199	0.1765	0.403	0.029
April+June	*Lemonniera centrosphaera*	0.872	0.8793	0.876	0.001
	Tetracladium sp.	0.9103	0.7414	0.822	0.001
April+September	*Flagellospora fusarioides*	0.8511	0.7021	0.773	0.004
	Mycoarthris corallina	0.9516	0.383	0.604	0.003
August+September	*Campylospora chaetocladia*	1	0.2128	0.461	0.01
April+June+August	*Dimorphospora* sp.	0.994	0.7386	0.857	0.001
June+August+September	*Tetrachaetum elegans*	0.9637	0.7333	0.841	0.002

Table S6: Continuation

Hyphomycete ASVs					
Time point	Taxon	A	B	IndVal	p-value
April	ASV9 (*Filosporella annelidica*)	0.9158	0.8333	0.874	0.001
	ASV14 (*Lemonniera centrosphaera*)	0.8951	0.4667	0.646	0.001
	ASV47 (T*etracladium* sp.)	0.935	0.4333	0.637	0.002
	ASV79 (*Filosporella annelidica*)	0.7843	0.2667	0.457	0.015
	ASV142 (*Tetracladium marchalianum*)	0.8601	0.2333	0.448	0.035
	ASV279 (*Tetracladium* sp.)	1	0.1667	0.408	0.007
	ASV324 (*Mycoarthris corallina*)	1	0.1667	0.408	0.004
	ASV44 (*Flagellospora fusarioides*)	1	0.1333	0.365	0.022
September	ASV12 (*Tetracladium marchalianum*)	0.8129	0.7059	0.758	0.001
	ASV16 (*Tetracladium marchalianum*)	0.7854	0.5882	0.68	0.001
	ASV357 (*Triscelophorus monosporus*)	0.9199	0.1765	0.403	0.027
April+June	ASV50 (*Tetracladium* sp.)	0.878	0.5	0.663	0.005
	ASV17 (*Lemonniera centrosphaera*)	0.968	0.2759	0.517	0.011
April+September	ASV116 (*Tetracladium marchalianum*)	0.9584	0.2766	0.515	0.001
	ASV54 (*Mycoarthris corallina*)	0.948	0.2766	0.512	0.014
August+September	ASV38 (*Campylospora chaetocladia*)	1	0.2128	0.461	0.009
April+June+August	ASV24 (*Dimorphospora* sp.)	0.9905	0.6932	0.829	0.001
	ASV34 (*Dimorphospora* sp.)	1	0.4773	0.691	0.001
April+June+September	ASV18 (*Flagellospora fusarioides*)	0.9228	0.52	0.693	0.013
	ASV56 (*Tetracladium marchalianum*)	0.9397	0.4133	0.623	0.001
June+August+September	ASV3 (*Tetrachaetum elegans*)	0.962	0.7333	0.84	0.001

Table S7: Results from the Indicator Taxa Analysis (IndVal) for the treatments of the study. A refers to the proportion that one taxon or an amplicon sequence variant (ASV) was found in a single treatment. B refers to the proportion of one taxon or ASV occurring in all samples of that treatment. Only taxa or ASVs that were significantly related to one or several treatments are given in this table. The taxa names added to the ASVs in brackets were assigned using UNITE (Abarenkov et al., 2020) database.

Hyphomycete taxa identified with morphological identification

treatment	Taxon	A	B	IndVal	p-value
forest	*Tetracladium* sp.	0.836	0.1111	0.305	0.045
viticulture	*Lunulospora sp.*	0.9437	0.3714	0.592	0.001
	Lunulospora curvula	0.9538	0.1714	0.404	0.018
forest+transplant	*Tetrachaetum elegans*	0.9311	0.8169	0.872	0.001
	Articulospora tetracladia	0.9294	0.5352	0.705	0.002
	Tricladium chaetocladium	0.9805	0.3099	0.551	0.015

Hyphomycete taxa identified via metabarcoding

treatment	Taxon	A	B	IndVal	p-value
viticulture	*Triscelophorus monosporus*	0.9825	0.1714	0.41	0.002
forest+transplant	*Tricladium splendens*	0.9469	0.9143	0.93	0.001
	Dimorphospora sp.	0.8404	0.8	0.82	0.001
	Filosporella annelidica	0.9033	0.6857	0.787	0.006

Hyphomycete ASVs

treatment	Taxon	A	B	IndVal	p-value
viticulture	ASV47 (T*etracladium* sp.)	0.7827	0.4	0.56	0.008
	ASV55 (*Anguillospora longissima*)	0.9144	0.2286	0.457	0.002
	ASV142 (*Tetracladium marchalianum*)	0.803	0.2571	0.454	0.005
	ASV357 (*Triscelophorus monosporus*)	0.9825	0.1714	0.41	0.003
	ASV268 (*Gibberella pulicaris*)	1	0.1429	0.378	0.006
	ASV279 (*Tetracladium* sp.)	1	0.1429	0.378	0.005
forest+transplant	ASV7 (*Tricladium splendens*)	0.9466	0.9143	0.93	0.001
	ASV24 (*Dimorphospora* sp.)	0.8907	0.7571	0.821	0.001
	ASV9 (*Filosporella annelidica*)	0.9276	0.5857	0.737	0.007
	ASV69 (*Tetrachaetum elegans*)	1	0.1571	0.396	0.05
transplant+viticulture	ASV5 (*Anguillospora longissima*)	0.8712	0.7857	0.827	0.001
	ASV11 (*Lemonniera centrosphaera*)	0.811	0.4143	0.58	0.049

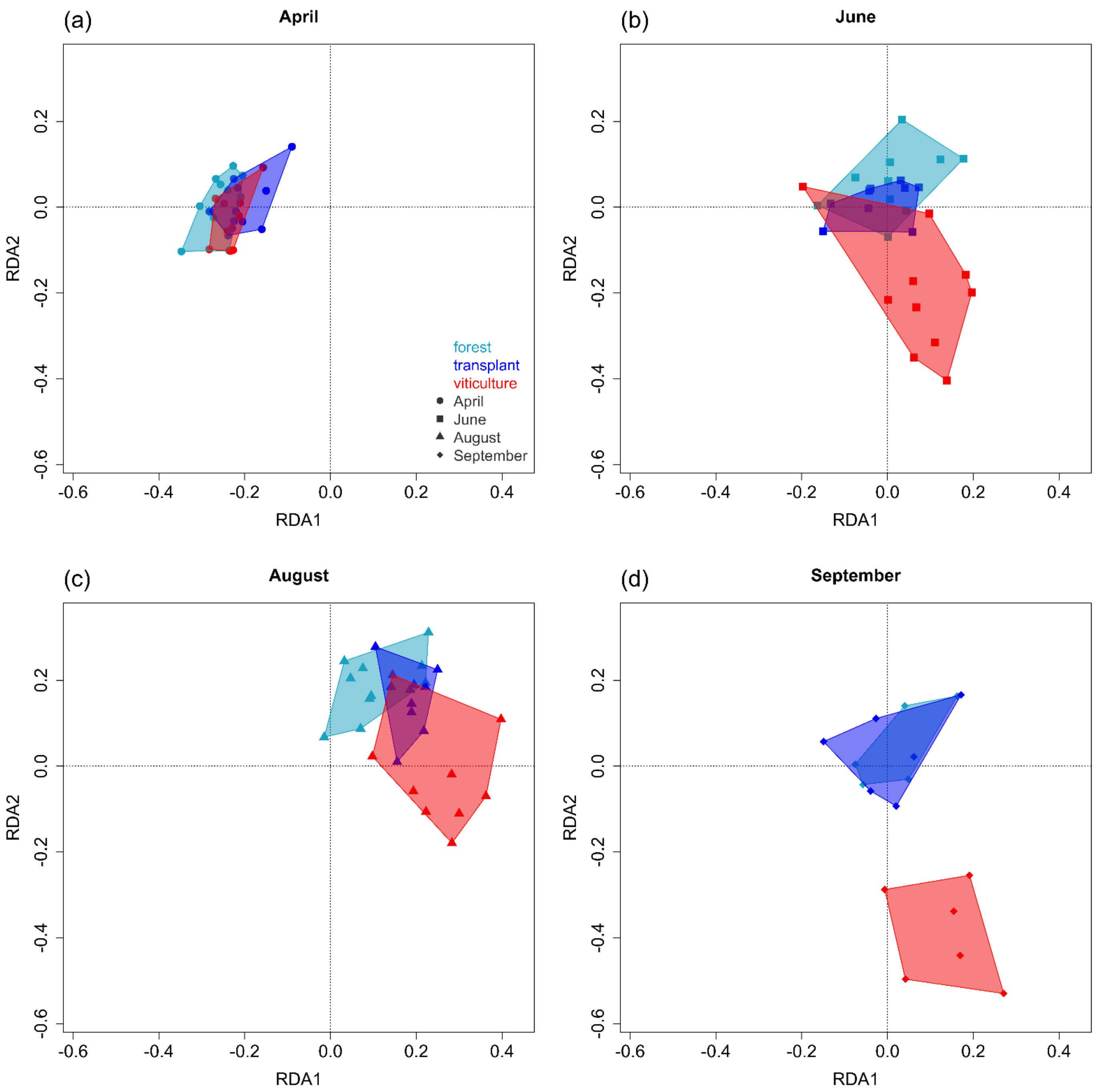

Figure S7: Morphologically identified hyphomycete communities across treatments for single time points. Each point within the redundancy analysis (RDA) represents the community of a single stream-treatment-time point combination (technical replicates were pooled before identification). The colours represent the treatments (see a, April), and the shape represents the time point which is in accordance with Figure 2. The p-values are provided in Table S5. During the September time point, leaf bags were only deployed at six of the ten streams due to droughts and for a deployment time of two instead of three weeks (Table S2).

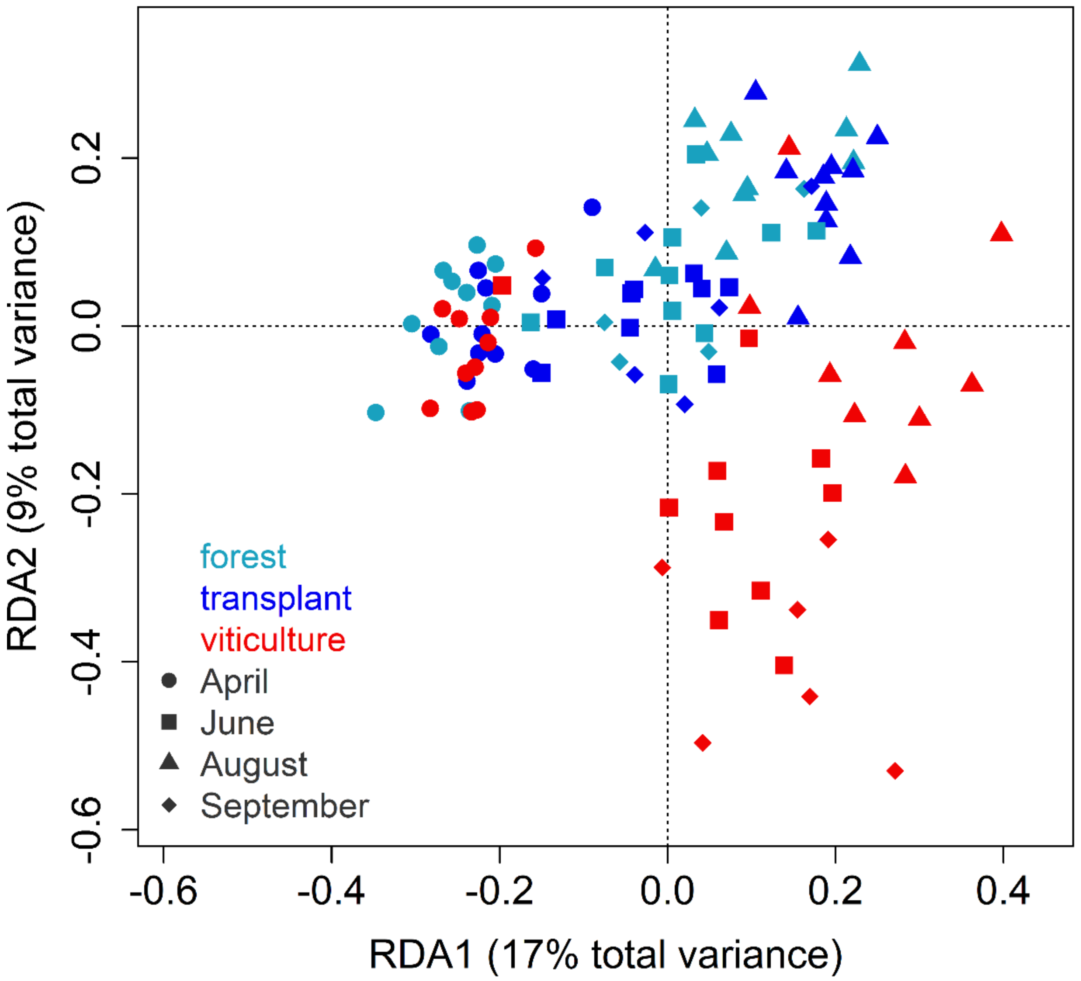

Figure S8: Morphologically identified hyphomycete communities across different treatments and time points. Reproduction of Figure 2 without polygons to enhance visibility of individual samples. Each point within the redundancy analysis (RDA) represents the community of a single stream-treatment-time point combination (technical replicates were pooled before identification). The colours represent the different treatments, and the shapes represents the different time point. The p-values are provided in Table S5.

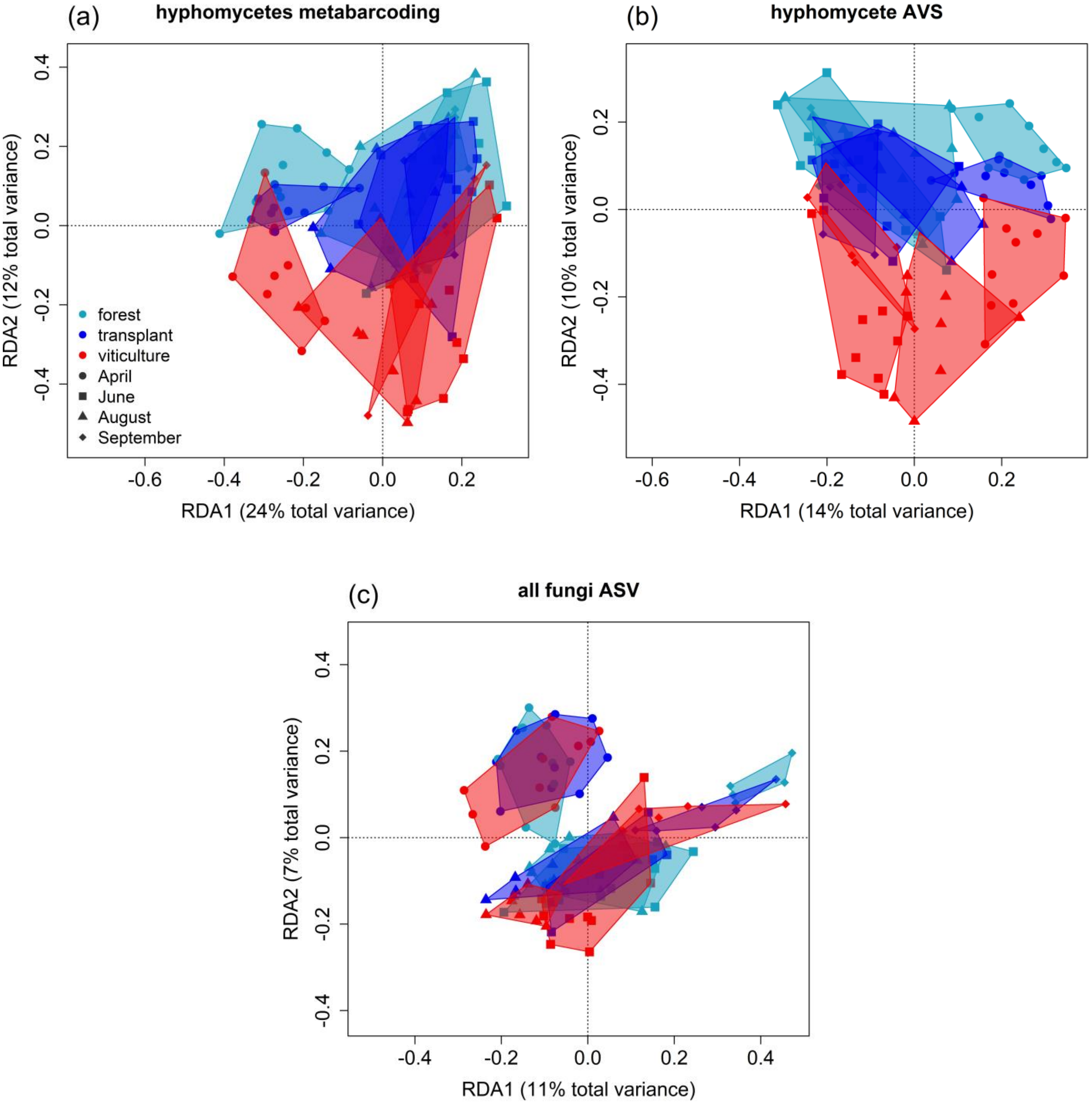

Figure S9: Communities of hyphomycetes on species and genus (a) and amplicon sequence variants (ASV) level (b) as well as the whole fungal community (c) identified with metabarcoding across treatments and time points. Each point within the redundancy analysis (RDA) represents the community of one stream-treatment-time point combination (technical replicates were pooled before analysis). The colours refer to the treatment, while the shape refers to the time point (see a). The p-values are provided in Table S5. During the September time point, leaf bags were only deployed at six of the otherwise ten streams due to droughts (Table S2)

Supporting text S1 – results on the whole and hyphomycete community using metabarcoding

In contrast to the hyphomycete communities identified morphologically, the whole fungal as well as the hyphomycete communities on species and genus as well as ASV level identified via metabarcoding responded significantly to the treatment and the time points, but not to the interaction of these two factors (Figure S9, Table S5). In accordance with the morphological data, the communities derived from metabarcoding data of the April time point clearly differed from those of later time points (Figure 2, Figure S9), which can also be explained by higher taxa or ASV richness (Figure S6), though the difference is not as pronounced as in the morphological data. The communities of the viticultural treatment identified with metabarcoding deviated already in April from those of the forest and transplant treatment, which is in contrast to the communities identified morphologically (Figure S9). For the three following time points, the metabarcoding and morphological data show similar patterns with communities from the forest and transport treatment, displaying strong overlaps and deviations of the viticultural treatment. This deviation is smallest when considering the whole fungal community and not only aquatic hyphomycetes (Figure S9, Table S5). This stronger overlap of the whole fungal community is supported by the fact that no ASV was identified as an indicator taxon to one or several of the treatments (Table S7), showing their cosmopolitan nature.

References

Abarenkov, K., Zirk, A., Piirmann, T., Pöhonen, R., Ivanov, F., Nilsson, R.H., Kõljalg, U., 2020. UNITE general FASTA release for Fungi. UNITE Community. https://doi.org/10.15156/BIO/786368

Baselga, A., Orme, C.D.L., 2012. betapart: an R package for the study of beta diversity. Methods in Ecology and Evolution 3, 808–812. https://doi.org/10.1111/j.2041-210X.2012.00224.x

De Cáceres, M., Legendre, P., Moretti, M., 2010. Improving indicator species analysis by combining groups of sites. Oikos 119, 1674–1684. https://doi.org/10.1111/j.1600-0706.2010.18334.x

European environmental agency, 2019. Corine Land Cover (CLC) 2018, Version 20 [WWW Document]. URL https://land.copernicus.eu/pan-european/corine-land-cover/clc2018 (accessed 10.15.19).

Lenth, R.V., Bolker, B., Buerkner, P., Giné-Vázquez, I., Herve, M., Jung, M., Love, J., Miguez, F., Riebl, H., Singmann, H., 2023. emmeans: Estimated Marginal Means, aka Least-Squares Means.

Tennekes, M., Nowosad, J., Gombin, J., Jeworutzki, S., Russell, K., Zijdeman, R., Clouse, J., Lovelace, R., Muenchow, J., 2022. tmap: Thematic Maps.
